# Necrosis by sodium overload: a potential mechanism for renal diseases associated with mitochondrial dysfunction

**DOI:** 10.1038/s41420-026-03111-0

**Published:** 2026-04-11

**Authors:** Quanhai Liu, Jiawei Lai, Jiancang Ma, Fangshi Xu

**Affiliations:** 1 Department of Urology, Shaanxi Provincial People’s Hospital, Xi’an, China; 2 Department of Vascular Surgery, The Second Affiliated Hospital of Xi’an Jiaotong University, Xi’an, China

**Keywords:** Cell death, Pathogenesis

## Introduction

Mitochondrial dysfunction is widely recognized as a key pathogenic feature of renal diseases [1]. Recently, an excellent study published in ‘Cell Death Discovery’ revealed a novel mechanism for kidney injury, that the ERK signaling pathway mediated mitophagy [2]. Coincidentally, an innovative mode of cell death named ‘necrosis by sodium overload’ (NECSO) was first identified in February 2025, which is closely associated with mitochondrial dysfunction and has garnered great interest [3]. In spite of the complicated nature of kidney disease pathogenesis, containing abnormal oxidative stress, immune response and inflammatory reaction, considerable research demonstrated that programmed cell death (PCD) induced by a variety of factors is the pivotal process in kidney damage and disease progression [4]. Being a new member of PCD, NECSO provides us with substantial inspiration, hinting that it could be an essential pathogenic mechanism hidden inside kidney diseases, which opens up new avenues for therapeutic target development.

## Tight associations between renal diseases and mitochondrial dysfunction

Mitochondria are essential for cellular energy balance. When this homeostasis is broken by mitochondria dysfunction, it can disturb the function of many organs, especially those rich in mitochondria, and the kidneys are precisely the victims of this pathological process. A wide range of studies have demonstrated that mitochondrial dysfunction mediates the progression of acute kidney injury (AKI) and chronic kidney disease (CKD), and also affects the repair process following kidney injury [5].

Mechanistically, mitochondrial quality control is the prominent pathway to realize renal diseases, which incorporates monitoring mitochondrial morphology, quantity, and quality [6]. Since the damaged mitochondria failed to generate sufficient energy, thereby leading to renal injury and impaired renal function. For instance, a recent study demonstrated that p53-induced PGC-1α downregulation can lead to maladaptive kidney repair [7]. More precisely, PGC-1α is required for producing new mitochondria, its deletion can extremely hinder mitochondrial biogenesis, resulting in energy deficiency and excessive oxidative stress [8]. Hence, transcriptional inhibition of PGC-1α caused by activation of p53 can hinder kidney repair after CKD [7]. Moreover, the abnormalities in mitochondrial quality can increase mitochondrial permeability, which activates apoptotic and autophagy pathways via the leakage of cytochrome c [9]. Under physiological states, mitochondria autophagy can maintain the stability of mitochondria quality through selectively scavenging excess and defective mitochondria [6]. However, excessive mitochondrial autophagy exacerbates damage in renal tubular epithelial cell through PINK1-dependent or -independent pathways [10].

Collectively, mitochondria dysfunction and consequent cell death are pivotal driving forces in the onset of kidney diseases. Mitochondria-target therapy has the potential to be a promising approach for protecting kidney health [11]. For instance, MitoQ a commonly-used antioxidant can alleviate diabetic kidney disease through targeting mitophagy mediated by XIAP-ULK1 axis [12]. Hsu SN *et al*. have also found that MitoQ can preserve bone health in patients with renal osteodystrophy (ROD) through block mitophagy [13].

## Mitochondrial dysfunction in kidney diseases: a trigger for diverse cell death processes

A tremendous amount of research highlighted the significant impact of mitochondrial dysfunction on health maintenance and disease progression [14]. As a central phase in multiple biological processes such as inflammation, injury, and repair, programmed cell death (PCD) is highly vulnerable to induction by mitochondrial dysfunction [15]. An essential foundation for the strong link between PCD and mitochondrial dysfunction is the reliance on ROS that arises from cellular energy imbalance [16]. Mitochondria are primarily responsible for energy production in cells through the process of aerobic oxidative phosphorylation. If mitochondrial function is compromised, the electron transport chain (ETC) will leak electrons that merge with oxygen molecules, producing superoxide anions, the main form of ROS [17]. Excessive ROS leads to damage in biological macromolecules, including DNA and proteins, and initiates the intrinsic apoptosis pathway via mitochondrial permeability transition and the suppression of anti-apoptotic proteins [18]. Cell apoptosis, in this regard, is a consequence of mitochondrial dysfunction.

As research advances, various patterns of programmed cell death (PCD), beyond apoptosis, have been demonstrated to be driven by mitochondrial dysfunction. Accordingly, this dysfunction also plays a role in the progression of kidney diseases through diverse cellular mechanisms. For instance, ACSL4/GPX4 and FSP1 axes can significantly improve the oxalate-induced renal injury [19]. NCOA7 suppresses the advancement of kidney cancer by promoting autophagy and lipid metabolism via its interaction with V-ATPase [20]. Indeed, different mitochondrial-induced PCD pathways have overlapping aspects, such as the accumulation of ROS, changes in mitochondrial morphology, and the interruption of ATP production. These traits were also found in the novel mode of cell death NECSO [3], highlighting the closed associations of this new pathogenic mechanism with mitochondrial dysfunction.

## Introducing a novel cell death linked to mitochondria dysfunction: necrotic death caused by sodium overload

Programmed cell death (PCD) is the key hub for regulating multiple biological processes. Recently, a novel mode of PCD has attracted widespread attention, termed ‘necrosis by sodium overload’ (NECSO) [3]. As shown in the paper, the activation of TRPM4 characterizes a distinct form of necrosis that can be triggered by either the anticancer compound Necrocide 1 (NC1) or the deletion of cellular energy. As a cation channel that is nonselective, TRPM4 activation resulted in a considerable influx of sodium and an efflux of potassium, with stable levels of calcium, magnesium, and ferrous ions, showing that sodium overload triggered necrotic cell death induced by NC1. Given that TRPM4’s channel function is regulated by voltage and energy deficiency will cause its overactivation [21], therefore, mitochondrial dysfunction, as a dominant cause of energy deficiency, was tightly associated with NECSO. Clearly, NECSO is another new member of the large family of mitochondrial dysfunction induced PCD.

## Functional alteration of ETC under solidum overload

As a critical hallmark of NECSO, sodium overload interferes with electron transport chain (ETC) function in a multi-target manner. This effect possesses cascade-amplifying and self-reinforcing characteristics, ultimately leading to a marked decline in oxidative phosphorylation (OXPHOS) efficiency and a crisis of cellular energy. When sodium overload occurs, it leads to sodium-calcium exchanger (NCX) dysfunction and restricted intracellular calcium efflux, resulting in calcium accumulation and the loss of calcium homeostasis [22]. This has profound impacts on the ETC function and OXPHOS process. First, intracellular imbalance of calcium and sodium ions will lead to conformational changes in ETC complex I (NADH dehydrogenase), reducing the affinity of the coenzyme Q binding site and thereby impairing electron transport [23]. Second, the accumulation of sodium can activate mitochondrial phospholipase A2 (PLA2) [24], disrupting the cardiolipin microenvironment. This further leads to structural damage of ETC complex III/IV (ubiquinone-cytochrome c reductase), impairing the efficiency of electron transfer from ubiquinone to cytochrome C. Third, the calcium excess resulted from sodium overload can inhibit the activity of ETC complex IV (cytochrome c oxidase) through competitively binding to its heme a3-CuB binuclear center, thereby inhibiting the reduction of oxygen to water and leading to a blockage on the electron flow [25]. Clearly, sodium overload disrupts mitochondrial oxidative phosphorylation and ETC function by altering ionic homeostasis.

## NECSO: a possible disease-causing mechanism for kidney disorders

**Fig. 1:**
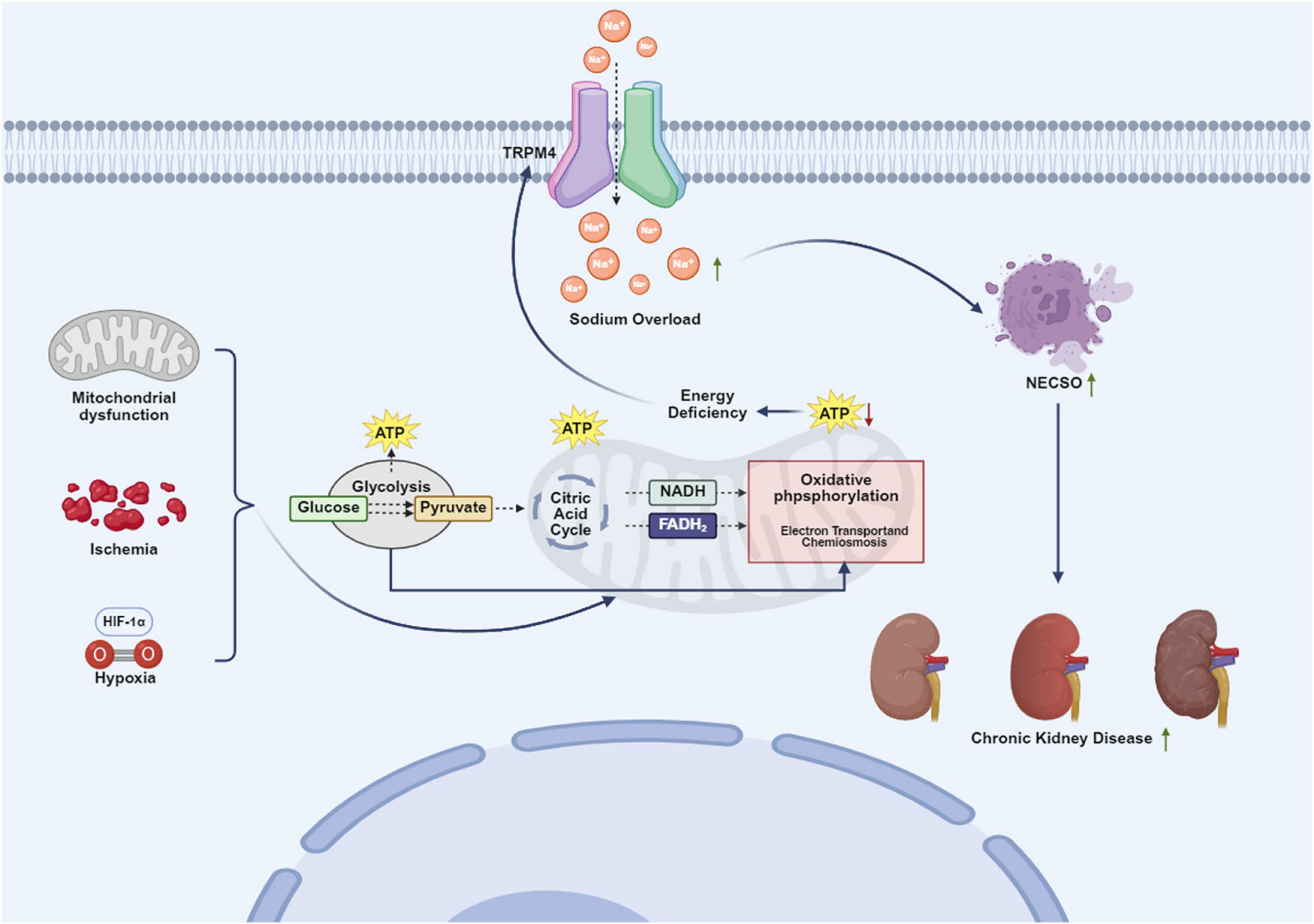
Potential interaction between NECSO and mitochondrial dysfunction. Mitochondrial dysfunction commonly leads to ATP deletion, thereby activating TRPM4. Active TRPM4 enhances the influx of sodium ions, eventually leading to sodium overload and NECSO. NECSO is a potential pathogenic mechanism of chronic kidney disease. NECSO, necrotic cell death through sodium overload.

As a novel type of cell death, NECSO greatly broadens our understanding of the diseases associated with mitochondrial dysfunction. Given that tight interaction between mitochondrial dysfunction, kidney diseases and cell death, then, a crucial question, is NECSO also involved in the pathogenesis of kidney diseases? Available evidence confirmed that ischemia and hypoxia are closely associated with TRPM4 function, while the former is also a critical driving factor in the pathophysiological process of CKD [26]. Based on existing research findings, it is speculated that CKD may yield the activation of TRPM4, ultimately inducing the occurrence of NECSO. First, ischemia and hypoxia cause marked cellular depolarization due to the activation of the nonselective cation channel (NC Ca-ATP) [27]. Since TRPM4 activity is voltage-dependent, the depolarization will significantly increase the probability of channel opening. Second, increased intracellular calcium is observed during ischemia-reperfusion injury [28], which further activates TRPM4 channel due to its high sensitivity to calcium. Third, TRPM4 expression is essential for the manifestation of some ischemia and hypoxia-related pathology [29]. Gerzanich V et al. have demonstrated that TRPM4 was heavily upregulated after spinal cord injury (SCI), but block of TRPM4 significantly reduced the secondary hemorrhage of SCI [29]. Fourth, sodium overload is essential for NECSO occurrence, while the concentration of sodium ions within the renal tubular epithelial cells typically tends to increase in CKD [30]. A classic example is the application of gliflozins in CKD, a sodium-glucose cotransporter 2 (SGLT2) inhibitor. Available evidence has demonstrated that gliflozins can protect renal cells through decreasing the load of sodium in macula densa [31]. Decreasing intracellular sodium concentration not only inhibits renal cell apoptosis, but also accelerates the flow within the glomerulus [31]. Moreover, TRPM4 inhibitors can substantially alleviate cell death induced by hypoxia, particularly in relation to neurodegeneration [21, 32]. Altogether, whether the activation of TRPM4 status or the sodium imbalance indicates that NECSO may participate in the pathogenesis of kidney diseases (Fig. 1).

## Therapeutic future of dihydropyridine calcium channel blockers

As the commonly-used antihypertensive drugs, dihydropyridine calcium channel blockers (DHP CCBs) are used to treat CKD through its potential inhibitory effects on NECSO [33]. Mechanistically, dihydropyridine can block L-type calcium channels and significantly reduce the concentration of intracellular calcium ion [34]. Activation of TRPM4 is highly dependent on intracellular calcium accumulation [35]. Studies have discovered that TRPM4 is significantly activated when the intracellular calcium concentration reaches 100 μM or higher [36]. Therefore, dihydropyridine can markedly reduce the ‘calcium signal’ required to trigger TRPM4 opening by blocking L-type calcium channels. Of note, DHP CCBs have lack effects on reducing proteinuria despite its good efficacy in reducing systemic hypertension [37]. This indicated that the preservation of renal function may rely on more specific CCBs to more precisely inhibit the activation of TRPM4.

## Future directions

There are several promising paths worth investigating to push this field forward. First, special focus should be given to the role of TRPM4 and cellular sodium levels to ensure the roles of NECSO in kidney disease are not overlooked. Consider the potential for NECSO to appear when the pathogenic process involves a disrupted sodium ion channel or a lack of energy. Secondly, NECSO may interact with other types of cell death. The major form of cell death resulting from mitochondrial dysfunction, apoptosis, operates as a collaborator rather than a single-threaded regulator. For instance, TRPM2 can give a remission on acute kidney injury (AKI) induced by cisplatin through modulating autophagy and apoptosis [38]. Thus, it’s evident that various PCD types interact within a network. Thirdly, NECSO offers new and promising therapeutic approaches. If the pathogenic effects of NECSO on kidney diseases could be confirmed, targeting TRPM4 could serve as an effective strategy to protect against renal function loss. Recently, the core binding sites for small molecule inhibitors targeting TRPM4 have been identified, which greatly contributes to drug research and development [39].

## Conclusions

Extensive evidence has confirmed the pivotal roles of mitochondrial homeostasis in kidney diseases. Due to a sophisticated network across mitochondrial dysfunction, cell death and renal disorder, with the arrival of NECSO, a viable and promising therapeutic method is available, focusing on TRPM4-mediated sodium overload. Future research highlighting TRPM4 status and sodium content in cells will aid in revealing this novel mechanism associated with kidney diseases.

## Availability of data and materials

Data sharing is not applicable to this article as no new data were created or analyzed in this study.

## Supplementary information

iThenticate plagiarism detection
