# Supplementary material for: Necrosis by sodium overload: a potential mechanism for renal diseases associated with mitochondrial dysfunction
Source: Cell Death Discov. 2026 Apr 11;12:226. doi: 10.1038/s41420-026-03111-0 (PMC13184122; doi:10.1038/s41420-026-03111-0)
Supplement: Supplementary file 1 — iThenticate plagiarism detection [file 41420_2026_3111_MOESM1_ESM.pdf]

none none

Necrosis by sodium overload: A potential mechanism for renal diseases associated with mitochondrial dysfunction

Institutions

Document Details

Submission ID

trn:oid:::3618:131569452

Submission Date

Mar 15, 2026, 10:26 AM GMT+8

Download Date

Mar 15, 2026, 10:29 AM GMT+8

File Name

1.docx

File Size

799.5 KB

10 Pages

3,029 Words

18,382 Characters

15% Overall Similarity

The combined total of all matches, including overlapping sources, for each database.

Filtered from the Report

- Bibliography

Match Groups

16 Not Cited or Quoted 8%
Matches with neither in-text citation nor quotation marks

14 Missing Quotations 7%
Matches that are still very similar to source material

0 Missing Citation 0%
Matches that have quotation marks, but no in-text citation

1 Cited and Quoted 0%
Matches with in-text citation present, but no quotation marks

Top Sources

3% Internet sources
15% Publications
0% Submitted works (Student Papers)

Integrity Flags

0 Integrity Flags for Review

No suspicious text manipulations found.

Our system's algorithms look deeply at a document for any inconsistencies that would set it apart from a normal submission. If we notice something strange, we flag it for you to review.

A Flag is not necessarily an indicator of a problem. However, we'd recommend you focus your attention there for further review.

Match Groups

- **16** Not Cited or Quoted 8%
  Matches with neither in-text citation nor quotation marks
- **14** Missing Quotations 7%
  Matches that are still very similar to source material
- **0** Missing Citation 0%
  Matches that have quotation marks, but no in-text citation
- **1** Cited and Quoted 0%
  Matches with in-text citation present, but no quotation marks

Top Sources

3% Internet sources
15% Publications
0% Submitted works (Student Papers)

Top Sources

The sources with the highest number of matches within the submission. Overlapping sources will not be displayed.

1	Publication	
	Danrui Cai, Hangyu Fu, Jiancang Ma, Fangshi Xu. "Necrotic cell death through sod...	10%
2	Internet	
	www.imrpress.com	<1%
3	Publication	
	Zeid J. Khitan, Antonios H. Tzamaloukas, Sergey Brodsky, Joseph I. Shapiro. "Dihy...	<1%
4	Internet	
	www.mdpi.com	<1%
5	Publication	
	Kok Poh Loh, Gandi Ng, Chye Yun Yu, Chee Kong Fhu, Dejie Yu, Rudi Vennekens, B...	<1%
6	Publication	
	Yuhui Qiao, Jianghuang Wang, Bohong Wang, Hong Zhou, Qianlin Ni, Wan Fu, Zep...	<1%
7	Internet	
	pmc.ncbi.nlm.nih.gov	<1%
8	Internet	
	scienmag.com	<1%
9	Internet	
	www.frontiersin.org	<1%
10	Internet	
	www2.mdpi.com	<1%

11 Publication

Yonggang Dai, Hongya Wang, Wei Wang, Xuewei Zhuang. "Multidimensional pan ... <1%

Article Type: Comment

Necrosis by sodium overload: A potential mechanism for renal diseases associated with mitochondrial dysfunction

Introduction:

Mitochondrial dysfunction is widely recognized as a key pathogenic feature of renal diseases (1). Recently, an excellent study published in ‘’Cell Death Discovery’ revealed a novel mechanism for kidney injury, that the ERK signaling pathway mediated mitophagy (2). Coincidentally, an innovative mode of cell death named ‘necrosis by sodium overload’ (NECSO) was first identified in February 2025, which is closely associated with mitochondrial dysfunction and has garnered great interest (3). In spite of the complicated nature of kidney disease pathogenesis, containing abnormal oxidative stress, immune response and inflammatory reaction, considerable research demonstrated that programmed cell death (PCD) induced by a variety of factors is the pivotal process in kidney damage and disease progression (4). Being a new member of PCD, NECSO provides us with substantial inspiration, hinting that it could be an essential pathogenic mechanism hidden inside kidney diseases, which opens up new avenues for therapeutic target development.

Tight associations between renal diseases and mitochondrial dysfunction

Mitochondria are essential for cellular energy balance. When this homeostasis is broken by mitochondria dysfunction, it can disturb the function of many organs, especially those rich in mitochondria, and the kidneys are precisely the victims of this pathological process. A wide range of studies have demonstrated that mitochondrial dysfunction mediates the progression of acute kidney injury (AKI) and chronic kidney disease (CKD), and also affects the repair process following kidney injury (5).

Mechanistically, mitochondrial quality control is the prominent pathway to realize renal diseases, which incorporates monitoring mitochondrial morphology, quantity, and

quality (6). Since the damaged mitochondria failed to generate sufficient energy, thereby leading to renal injury and impaired renal function. For instance, a recent study demonstrated that p53-induced PGC-1α downregulation can lead to maladaptive kidney repair (7). More precisely, PGC-1α is required for producing new mitochondria, its deletion can extremely hinder mitochondrial biogenesis, resulting in energy deficiency and excessive oxidative stress (8). Hence, transcriptional inhibition of PGC-1α caused by activation of p53 can hinder kidney repair after CKD (7). Moreover, the abnormalities in mitochondrial quality can increase mitochondrial permeability, which activates apoptotic and autophagy pathways via the leakage of cytochrome c (9). Under physiological states, mitochondria autophagy can maintain the stability of mitochondria quality through selectively scavenging excess and defective mitochondria (6). However, excessive mitochondrial autophagy exacerbates damage in renal tubular epithelial cell through PINK1-dependent or -independent pathways (10).

Collectively, mitochondria dysfunction and consequent cell death are pivotal driving forces in the onset of kidney diseases. Mitochondria-target therapy has the potential to be a promising approach for protecting kidney health (11). For instance, MitoQ a commonly-used antioxidant can alleviate diabetic kidney disease through targeting mitophagy mediated by XIAP-ULK1 axis (12) . Hsu SN *et al.* have also found that MitoQ can preserve bone health in patients with renal osteodystrophy (ROD) through block mitophagy (13).

Mitochondrial dysfunction in kidney diseases: A trigger for diverse cell death processes

A tremendous amount of research highlighted the significant impact of mitochondrial dysfunction on health maintenance and disease progression (14). As a central phase in multiple biological processes such as inflammation, injury, and repair, programmed cell death (PCD) is highly vulnerable to induction by mitochondrial dysfunction (15). An essential foundation for the strong link between PCD and mitochondrial dysfunction is the reliance on ROS that arises from cellular energy imbalance (16). Mitochondria are primarily responsible for energy production in cells

through the process of aerobic oxidative phosphorylation. If mitochondrial function is compromised, the electron transport chain (ETC) will leak electrons that merge with oxygen molecules, producing superoxide anions, the main form of ROS (17). Excessive ROS leads to damage in biological macromolecules, including DNA and proteins, and initiates the intrinsic apoptosis pathway via mitochondrial permeability transition and the suppression of anti-apoptotic proteins (18). Cell apoptosis, in this regard, is a consequence of mitochondrial dysfunction.

As research advances, various patterns of programmed cell death (PCD), beyond apoptosis, have been demonstrated to be driven by mitochondrial dysfunction. Accordingly, this dysfunction also plays a role in the progression of kidney diseases through diverse cellular mechanisms. For instance, ACSL4/GPX4 and FSP1 axes can significantly improve the oxalate-induced renal injury (19). NCOA7 suppresses the advancement of kidney cancer by promoting autophagy and lipid metabolism via its interaction with V-ATPase (20). Indeed, different mitochondrial-induced PCD pathways have overlapping aspects, such as the accumulation of ROS, changes in mitochondrial morphology, and the interruption of ATP production. These traits were also found in the novel mode of cell death NECSO (3), highlighting the closed associations of this new pathogenic mechanism with mitochondrial dysfunction.

Introducing a novel cell death linked to mitochondria dysfunction: Necrotic death caused by sodium overload

Programmed cell death (PCD) is the key hub for regulating multiple biological processes. Recently, a novel mode of PCD has attracted widespread attention, termed 'necrosis by sodium overload' (NECSO) (3). As shown in the paper, the activation of TRPM4 characterizes a distinct form of necrosis that can be triggered by either the anticancer compound Necrocide 1 (NC1) or the deletion of cellular energy. As a cation channel that is non-selective, TRPM4 activation resulted in a considerable influx of sodium and an efflux of potassium, with stable levels of calcium, magnesium, and ferrous ions, showing that sodium overload triggered necrotic cell death induced by NC1. Given that TRPM4's channel function is regulated by voltage and energy

deficiency will cause its overactivation (21), therefore, mitochondrial dysfunction, as a dominant cause of energy deficiency, was tightly associated with NECSO. Clearly, NECSO is another new member of the large family of mitochondrial dysfunction induced PCD.

Functional alteration of ETC under solidum overload

As a critical hallmark of NECSO, sodium overload interferes with electron transport chain (ETC) function in a multi-target manner. This effect possesses cascade-amplifying and self-reinforcing characteristics, ultimately leading to a marked decline in oxidative phosphorylation (OXPHOS) efficiency and a crisis of cellular energy. When sodium overload occurs, it leads to sodium-calcium exchanger (NCX) dysfunction and restricted intracellular calcium efflux, resulting in calcium accumulation and the loss of calcium homeostasis (22). This has profound impacts on the ETC function and OXPHOS process. First, intracellular imbalance of calcium and sodium ions will lead to conformational changes in ETC complex I (NADH dehydrogenase), reducing the affinity of the coenzyme Q binding site and thereby impairing electron transport (23). Second, the accumulation of sodium can activate mitochondrial phospholipase A2 (PLA2) (24), disrupting the cardiolipin microenvironment. This further leads to structural damage of ETC complex III/IV (ubiquinone-cytochrome c reductase), impairing the efficiency of electron transfer from ubiquinone to cytochrome C. Third, the calcium excess resulted from sodium overload can inhibit the activity of ETC complex IV (cytochrome c oxidase) through competitively binding to its heme a3-CuB binuclear center, thereby inhibiting the reduction of oxygen to water and leading to a blockage on the electron flow (25). Clearly, sodium overload disrupts mitochondrial oxidative phosphorylation and ETC function by altering ionic homeostasis.

NECSO: A possible disease-causing mechanism for kidney disorders

As a novel type of cell death, NECSO greatly broadens our understanding of the diseases associated with mitochondrial dysfunction. Given that tight interaction between mitochondrial dysfunction, kidney diseases and cell death, then, a crucial

question, is NECSO also involved in the pathogenesis of kidney diseases? Available evidence confirmed that ischemia and hypoxia are closely associated with TRPM4 function, while the former is also a critical driving factor in the pathophysiological process of CKD (26). Based on existing research findings, it is speculated that CKD may yield the activation of TRPM4, ultimately inducing the occurrence of NECSO. First, ischemia and hypoxia cause marked cellular depolarization due to the activation of the nonselective cation channel (NC Ca-ATP) (27). Since TRPM4 activity is voltage-dependent, the depolarization will significantly increase the probability of channel opening. Second, increased intracellular calcium is observed during ischemia-reperfusion injury (28), which further activates TRPM4 channel due to its high sensitivity to calcium. Third, TRPM4 expression is essential for the manifestation of some ischemia and hypoxia-related pathology (29). Gerzanich V et al. have demonstrated that TRPM4 was heavily upregulated after spinal cord injury (SCI), but block of TRPM4 significantly reduced the secondary hemorrhage of SCI (29). Fourth, sodium overload is essential for NECSO occurrence, while the concentration of sodium ions within the renal tubular epithelial cells typically tends to increase in CKD (30). A classic example is the application of gliflozins in CKD, a sodium-glucose cotransporter 2 (SGLT2) inhibitor. Available evidence has demonstrated that gliflozins can protect renal cells through decreasing the load of sodium in macula densa (31). Decreasing intracellular sodium concentration not only inhibits renal cell apoptosis, but also accelerates the flow within the glomerulus (31). Moreover, TRPM4 inhibitors can substantially alleviate cell death induced by hypoxia, particularly in relation to neurodegeneration (21, 32). Altogether, whether the activation of TRPM4 status or the sodium imbalance indicates that NECSO may participate in the pathogenesis of kidney diseases (Figure 1).

Therapeutic future of dihydropyridine calcium channel blockers

As the commonly-used antihypertensive drugs, dihydropyridine calcium channel blockers (DHP CCBs) are used to treat CKD through its potential inhibitory effects on NECSO (33). Mechanistically, dihydropyridine can block L-type calcium channels and significantly reduce the concentration of intracellular calcium ion (34). Activation of

TRPM4 is highly dependent on intracellular calcium accumulation (35). Studies have discovered that TRPM4 is significantly activated when the intracellular calcium concentration reaches 100 μM or higher (36). Therefore, dihydropyridine can markedly reduce the 'calcium signal' required to trigger TRPM4 opening by blocking L-type calcium channels. Of note, DHP CCBs have lack effects on reducing proteinuria despite its good efficacy in reducing systemic hypertension (37). This indicated that the preservation of renal function may rely on more specific CCBs to more precisely inhibit the activation of TRPM4.

Future directions

There are several promising paths worth investigating to push this field forward. First, special focus should be given to the role of TRPM4 and cellular sodium levels to ensure the roles of NECSO in kidney disease are not overlooked. Consider the potential for NECSO to appear when the pathogenic process involves a disrupted sodium ion channel or a lack of energy. Secondly, NECSO may interact with other types of cell death. The major form of cell death resulting from mitochondrial dysfunction, apoptosis, operates as a collaborator rather than a single-threaded regulator. For instance, TRPM2 can give a remission on acute kidney injury (AKI) induced by cisplatin through modulating autophagy and apoptosis (38). Thus, it's evident that various PCD types interact within a network. Thirdly, NECSO offers new and promising therapeutic approaches. If the pathogenic effects of NECSO on kidney diseases could be confirmed, targeting TRPM4 could serve as an effective strategy to protect against renal function loss. Recently, the core binding sites for small molecule inhibitors targeting TRPM4 have been identified, which greatly contributes to drug research and development (39).

Conclusions

Extensive evidence has confirmed the pivotal roles of mitochondrial homeostasis in kidney diseases. Due to a sophisticated network across mitochondrial dysfunction, cell death and renal disorder, with the arrival of NECSO, a viable and promising therapeutic method is available, focusing on TRPM4-mediated sodium overload. Future research highlighting TRPM4 status and sodium content in cells will aid in

revealing this novel mechanism associated with kidney diseases.

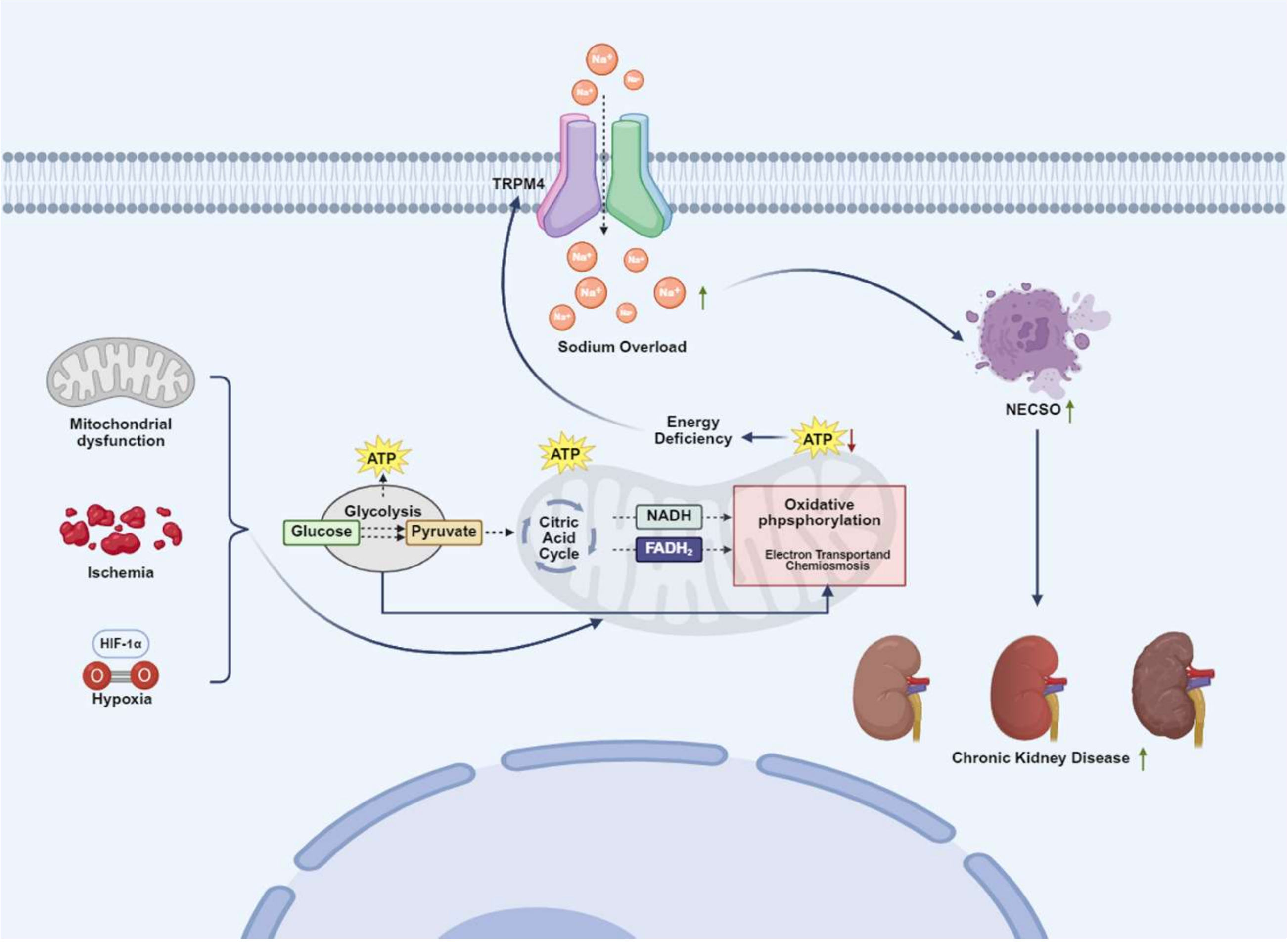

References:

1. Amador-Martínez I, Aranda-Rivera AK, Martínez-Castañeda MR, Pedraza-Chaverri J. Mitochondrial quality control and stress signaling pathways in the pathophysiology of cardio-renal diseases. Mitochondrion. 2025;84:102040.
2. Wang D, Li Y, Li G, Liu M, Zhou Z, Wu M, et al. Inhibition of PKC-δ retards kidney fibrosis via inhibiting cGAS-STING signaling pathway in mice. Cell death discovery. 2024;10(1):314.
3. Fu W, Wang J, Li T, Qiao Y, Zhang Z, Zhang X, et al. Persistent activation of TRPM4 triggers necrotic cell death characterized by sodium overload. Nature chemical biology. 2025.
4. Liu F, Yang Z, Li J, Wu T, Li X, Zhao L, et al. Targeting programmed cell death in diabetic kidney disease: from molecular mechanisms to pharmacotherapy. Molecular medicine (Cambridge, Mass). 2024;30(1):265.
5. Guo Y, Che R, Wang P, Zhang A. Mitochondrial dysfunction in the pathophysiology of renal diseases. American journal of physiology Renal physiology. 2024;326(5):F768-f79.
6. Tang C, Cai J, Yin XM, Weinberg JM, Venkatachalam MA, Dong Z. Mitochondrial quality control in kidney injury and repair. Nature reviews Nephrology. 2021;17(5):299-318.

7. Wang Y, Zeng Y, Fu Y, Liu Z, Hu X, Tang C, et al. Repression of peroxisome proliferation-activated receptor γ coactivator-1α by p53 after kidney injury promotes mitochondrial damage and maladaptive kidney repair. Kidney international. 2025;107(5):869-87.
8. Doke T, Susztak K. The multifaceted role of kidney tubule mitochondrial dysfunction in kidney disease development. Trends in cell biology. 2022;32(10):841-53.
9. Bauer TM, Murphy E. Role of Mitochondrial Calcium and the Permeability Transition Pore in Regulating Cell Death. Circulation research. 2020;126(2):280-93.
10. Su L, Zhang J, Gomez H, Kellum JA, Peng Z. Mitochondria ROS and mitophagy in acute kidney injury. Autophagy. 2023;19(2):401-14.
11. Thompson AD, Victor Santiago Raj P, Scholpa NE, Schnellmann RG. Repurposing mitochondria-targeted therapeutics for kidney diseases. Kidney international. 2025;107(4):617-27.
12. Hu H, Ji R, Hao Y, Liu Z, Yang J, Cao Y, et al. XIAP-ULK1-mediated mitophagy modulates carnitine metabolism to mitigate diabetic kidney disease. Autophagy. 2026;22(1):207-28.
13. Hsu SN, Stephen LA, Phadwal K, Dillon S, Carter R, Morton NM, et al. Mitochondrial dysfunction and mitophagy blockade contribute to renal osteodystrophy in chronic kidney disease-mineral bone disorder. Kidney international. 2025.
14. Aqeel A, Akram A, Ali M, Iqbal M, Aslam M, Rukhma, et al. Mechanistic insights into impaired β-oxidation and its role in mitochondrial dysfunction: A comprehensive review. Diabetes research and clinical practice. 2025;223:112129.
15. Ravindran R, Gustafsson Å B. Mitochondrial quality control in cardiomyocytes: safeguarding the heart against disease and ageing. Nature reviews Cardiology. 2025.
16. Kageyama Y, Okura S, Sukigara A, Matsunaga A, Maekubo K, Oue T, et al. The Association Among Bipolar Disorder, Mitochondrial Dysfunction, and Reactive Oxygen Species. Biomolecules. 2025;15(3).
17. Bochkova ZV, Baizhumanov AA, Yusipovich AI, Morozova KI, Nikelshparg EI, Fedotova AA, et al. The flexible chain: regulation of structure and activity of ETC complexes defines rate of ATP synthesis and sites of superoxide generation. Biophysical reviews. 2025;17(1):55-88.
18. An X, Yu W, Liu J, Tang D, Yang L, Chen X. Oxidative cell death in cancer: mechanisms and therapeutic opportunities. Cell death & disease. 2024;15(8):556.
19. Ye K, Lan R, Chen Z, Lai K, Song Y, Li G, et al. Roles of ACSL4/GPX4 and FSP1 in oxalate-induced acute kidney injury. Cell death discovery. 2025;11(1):279.
20. Wang J, Luo H, He Q, Shao H, Cai X, Cao Y, et al. NCOA7 inhibits renal cancer progression by inducing autophagy and lipid metabolism through V-ATPase interaction. Cell death discovery. 2025;11(1):471.
21. Yan J, Bengtson CP, Buchthal B, Hagenston AM, Bading H. Coupling of NMDA receptors and TRPM4 guides discovery of unconventional neuroprotectants. Science (New York, NY). 2020;370(6513).
22. DiPolo R, Beaugé L. Sodium/calcium exchanger: influence of metabolic regulation on ion carrier interactions. Physiological reviews. 2006;86(1):155-203.
23. Chavda V, Lu B. Reverse Electron Transport at Mitochondrial Complex I in Ischemic Stroke, Aging, and Age-Related Diseases. Antioxidants (Basel, Switzerland). 2023;12(4).

24. Glaser KB, Mobilio D, Chang JY, Senko N. Phospholipase A2 enzymes: regulation and inhibition. Trends in pharmacological sciences. 1993;14(3):92-8.

25. Pham L, Arroum T, Wan J, Pavelich L, Bell J, Morse PT, et al. Regulation of mitochondrial oxidative phosphorylation through tight control of cytochrome c oxidase in health and disease - Implications for ischemia/reperfusion injury, inflammatory diseases, diabetes, and cancer. Redox biology. 2024;78:103426.

26. Li C, Yu Y, Zhu S, Hu Y, Ling X, Xu L, et al. The emerging role of regulated cell death in ischemia and reperfusion-induced acute kidney injury: current evidence and future perspectives. Cell death discovery. 2024;10(1):216.

27. Chen M, Simard JM. Cell swelling and a nonselective cation channel regulated by internal Ca2+ and ATP in native reactive astrocytes from adult rat brain. The Journal of neuroscience : the official journal of the Society for Neuroscience. 2001;21(17):6512-21.

28. Leiva-Salcedo E, Riquelme D, Cerda O, Stutzin A. TRPM4 activation by chemically- and oxygen deprivation-induced ischemia and reperfusion triggers neuronal death. Channels (Austin, Tex). 2017;11(6):624-35.

29. Gerzanich V, Woo SK, Vennekens R, Tsymbalyuk O, Ivanova S, Ivanov A, et al. De novo expression of Trpm4 initiates secondary hemorrhage in spinal cord injury. Nature medicine. 2009;15(2):185-91.

30. Li CSZ, Yu B, Gao Q, Dong HL, Li ZL. The critical role of ion channels in kidney disease: perspective from AKI and CKD. Renal failure. 2025;47(1):2488139.

31. Girardi ACC, Polidoro JZ, Castro PC, Pio-Abreu A, Noronha IL, Drager LF. Mechanisms of heart failure and chronic kidney disease protection by SGLT2 inhibitors in nondiabetic conditions. American journal of physiology Cell physiology. 2024;327(3):C525-c44.

32. Chen B, Gao Y, Wei S, Low SW, Ng G, Yu D, et al. TRPM4-specific blocking antibody attenuates reperfusion injury in a rat model of stroke. Pflugers Archiv : European journal of physiology. 2019;471(11-12):1455-66.

33. Zhao HJ, Li Y, Liu SM, Sun XG, Li M, Hao Y, et al. Effect of calcium channels blockers and inhibitors of the renin-angiotensin system on renal outcomes and mortality in patients suffering from chronic kidney disease: systematic review and meta-analysis. Renal failure. 2016;38(6):849-56.

34. Tsien RW, Ellinor PT, Horne WA. Molecular diversity of voltage-dependent Ca2+ channels. Trends in pharmacological sciences. 1991;12(9):349-54.

35. Tian Y, Zheng J. The TRP channels serving as chemical-to-electrical signal converter. Physiological reviews. 2025;105(3):1033-74.

36. Ullrich ND, Voets T, Prenen J, Vennekens R, Talavera K, Droogmans G, et al. Comparison of functional properties of the Ca2+-activated cation channels TRPM4 and TRPM5 from mice. Cell calcium. 2005;37(3):267-78.

37. Sica D. Calcium channel blockers and the kidney. Clinical cornerstone. 2004;6(4):39-52.

38. Yu B, Jin L, Yao X, Zhang Y, Zhang G, Wang F, et al. TRPM2 protects against cisplatin-induced acute kidney injury and mitochondrial dysfunction via modulating autophagy. Theranostics. 2023;13(13):4356-75.

39. Ekundayo B, Arullampalam P, Gerber CE, Hämmerli AF, Guichard S, Boukenna M, et al. Identification of a binding site for small molecule inhibitors targeting human TRPM4. Nature communications. 2025;16(1):833.

Figure 1. Potential interaction between NECSO and mitochondrial dysfunction

Mitochondrial dysfunction commonly leads to ATP deletion, thereby activating TRPM4. Active TRPM4 enhances the influx of sodium ions, eventually leading to sodium overload and NECSO. NECSO is a potential pathogenic mechanism of chronic kidney disease. NECSO, necrotic cell death through sodium overload.
